# Reliability and Validity of the Korean Late-Life Function and Disability Instrument

**DOI:** 10.3390/healthcare9091200

**Published:** 2021-09-11

**Authors:** Da Sol Park, Hae Yean Park

**Affiliations:** 1 Department of Occupational Therapy, Jeonju Kijeon College, Jeonju 54989, Korea; otdasol@gmail.com; 2 Department of Occupational Therapy, College of Software Digital Healthcare Convergence, Yonsei University, Wonju 26493, Korea

**Keywords:** assessment, disability, evaluation, function, older adults

## Abstract

The purpose of this study was to develop the Korean version of the Late-Life Function and Disability Instrument (K-LLFDI) and verify its reliability and validity. Fifty community-dwelling older adults aged 65 years and above with independent mobility were surveyed. The reliability and validity of the instrument were verified. The overall cultural validity of 48 items was evaluated as very high (0.95), and only one item that was not appropriate was revised. The reliability of the remaining six domains was either high or very high. Internal consistency was high (α = 0.859) in the Disability component of the instrument and very high (α = 0.914) in the Function component. The factor loading for 42 out of 48 items was above 0.04. Overall, each component was well reflected by the sub-items. The K-LLFDI is expected to be instrumental in solving the rapidly growing problems of community-dwelling older adults.

## 1. Background

Late life is the last phase in an individual’s life cycle when physiological, biological, and mental functions change, and it is characterized by loss of individual abilities and social status. Worldwide, the average age of 65 years and older has been reported as a period in life characterized by low self-esteem, loneliness, and alienation due to role loss, when the subjective quality of life is affected more than any other stage of life [1,2]). Age is an important aspect of modern society, and it is estimated that the total cost of the population aged 65 years or older in Korea is 12.8%, which is expected to increase to 20% or more by 2026, making it an ultra-aged society [3].

Due to aging, older adults experience poor health conditions and dysfunction [4]. Dysfunction increases older adults’ dependence on others and negatively affects their quality of life [5]. These conditions have been closely related to Parkinson’s disease, heart disease, cancer, stroke, and dementia, which have been associated with prolonged medical use, long-term nursing home admission, death, and short life expectancy [6,7]. Older adults are also exposed to many neurological diseases such as multiple sclerosis [8]. Additionally, daily functioning at an older age has been directly related to a reduced ability to maintain an independent lifestyle and is used as an indicator of the effects of disease, disorders, and other health-related conditions [9,10].

Within the global health policy sector, the importance of older adult functional assessment has been highlighted, with the focus of gradually ensuring active retirement rather than simply extending life span [11]. Existing tools for assessing the function and disability of older adults include the Functional Independence Measure (FIM) and the Modified Barthel Index (MBI). The FIM has the advantage of periodically assessing patients’ performance to ensure that patients are properly treated and discharge plans are established [12]. The MBI is a highly sensitive instrument, which is simple and easy to score but not suitable for evaluating patients with poor overall performance such as stroke or brain damage because they do not know the state of their own general health or social condition [13]. Additionally, FIM and MBI are tools that focus on Activities of Daily Living (ADL) rather than function and disability. A previous study reported that there were few useful tools in the field of occupational therapy to evaluate functions and disabilities regarding the performance of everyday life in older adults [14]. Therefore, assessment tools focusing on function and disability for the elderly are now necessary. The Late-Life Function and Disability Instrument (LLFDI) is a standardized tool developed for assessing meaningful changes in the outcomes of both functional and disability categories by professors of Boston University’s Department of Occupational and Physical Therapy [15]. The LLFDI is based on the International Classification of Functioning (ICF), a model used by rehabilitation professionals in clinical work and research, and it is a self-reporting assessment of functions and disabilities for older adults living in the community [16]. The LLFDI has been translated into Hebrew, Swedish, French Canadian, and Brazilian Portuguese, and its reliability and validity have been verified [17,18,19,20]. This instrument is used in many countries around the world. In order to use assessment tools developed in other countries, not only do their reliability and validity need to be assessed, but also a cultural adaptation process is required, in which the tools are redesigned to suit the culture of a certain country [21]. This process has been reported in four Korean studies, and the method utilized was to translate the original tools developed into English.

The purpose of this study is to perform a cultural adaptation of the LLFDI to develop the Korean version of the Late-Life Function and Disability Instrument (K-LLFDI) and verify its reliability and validity. This tool can help older adults maintain a more independent lifestyle and improve their quality of life by properly assessing the functions and disabilities of this rapidly growing population. It could also be used as a basis for conducting further research on the functions and disabilities of older adults.

## 2. Methods

### 2.1. Subjects

This study was approved by the ethical board at Yonsei university [YUWIRB-1041849-201906-BM-085-01]. The study was conducted between December 2018 and February 2019 with participants aged 65 or older who lived in communities from the provinces of Gangwon and South Gyeongsang, Republic of Korea. Prior to the study, the purpose and method of the study were fully explained to the study participants, and only those who signed the informed consent were included. Criteria for selecting participants were older adults: (1) who were aged 65 years or more, (2) who could move independently without the help of other (Ambulation scores in MBI were 15 points), (3) without serious cognitive impairment, (4) and who understood the purpose of the study and agreed to participate. The criteria for determining serious cognitive impairment included not being diagnosed with dementia and people with MMSE scores of 24 or higher. Generally, if the MMSE is higher than 24 points, it is determined as a normal group. The total number of participants was 50, the average age was 79.24 ± 4, and average MMSE score was 25.25 ± 3. The evaluation tool did not have a score classification according to general information (BMI, smoking, marital status, etc.), so it was not further investigated. There were 11 men (22%) and 39 (78%) women. There was no dropout from the experiment.

### 2.2. Assessment Tool: Late-Life Function and Disability Instrument (LLFDI)

The LLFDI is a standardized evaluation tool developed in the United States in 2002 that assesses meaningful changes in two components (Disability and Function) in community-dwelling older adults [15]. There are 16 items within the disability component. Each item is numbered according to its order on the questionnaire, and it begins with “D” (for Disability) to differentiate it from the Function (F) items. Each dimension is marked by the appropriate letter: “a” for frequency and “b” for limitation. For example: “D1a. How often do you keep in touch with others?”, “D1b. To what extent do you feel limited in keeping in touch with others?”. The Disability component comprises 16 items in four domains classified as follows: frequency (social role (9 items: D1a, D2a, D3a, D5a, D6a, D9a, D11a, D12a, D14a), personal role (7 items: D4a, D7a, D8a, D10a, D13a, D15a, D16a)), limitation (instrumental role (12 items: D2b, D3b, D4b, D5b, D6b, D9b, D10b, D12b, D13b, D14b, D15b, D16b), and management role (4 items: D1b, D7b, D8b, D11b)). Both frequency and limitation have rating scale categories from 5 to 1. We used the scoring convention that the higher the scoring category, the more frequent the involvement in the activity and the less limited the person feels in completing activities. Therefore, the higher the score, the less disabled they are.

There are 32 items within the function component. Each item should be numbered according to its order on the questionnaire and begin with “F” (for Function) to differentiate it from the disability items. Thirty-two items in three domains are classified as follows: upper extremity function (7 items: F1, F3, F5, F6, F13, F16, F17), basic lower extremity function (14 items: F2, F10, F11, F12, F14, F15, F18, F21, F22, F23, F25, F26, F28, F31), and advanced lower extremity function (11 items: F4, F7, F8, F9, F19, F20, F24, F27, F29, F30, F32). The function component has a rating scale from 5 (None) to 1 (Cannot do). It uses the scoring convention that the higher the scoring category, the less difficulty the person has in doing activities.

LLFDI scores are provided as raw and scaled scores for a total of seven areas in the disability and function components. The scaled scores range from 0 to 100 points, and scores closer to 100 points indicate a higher level of function. In this study, we developed the Korean version of the LLFDI (K-LLFDI) by referring to the original English version by Jette et al. (2002) [15].

### 2.3. Procedure

**Figure 1:**
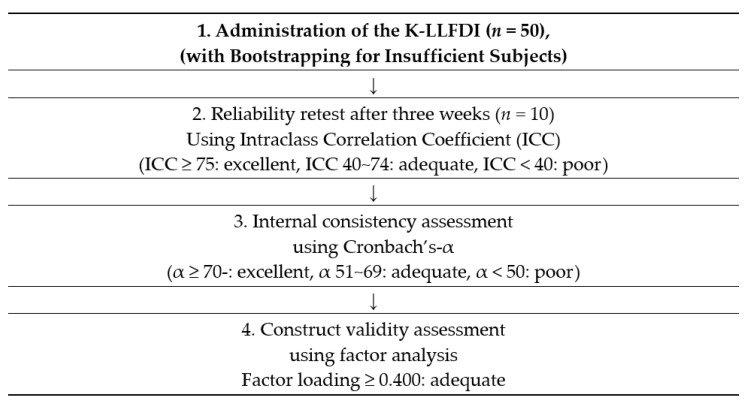
Research process.

This study was performed in three stages to develop the K-LLFDI and test its reliability and validity (Figure 1). The first stage was Translation and back-translation of the LLFDI. The second was the equation of item content validity for cultural adaptation of the LLFDI by 15 experts (professors, occupational therapists. Third, reliability and validity assessment of the K-LLFDI in community-dwelling older adults.

### 2.4. Stage 1: Translation and Back-Translation of the LLFDI

We first worked on translating the LLFDI into Korean. A researcher and a professional with a major in English literature translated the instrument, and later an occupational therapist who lived in an English-speaking country for more than 10 years performed the back-translation. During the translation and back-translation process, two occupational therapy professors who had research experience in English-speaking countries reviewed the results of both stages. The review includes differences in meaning and differences in grammar. Finally, the researchers made the final revisions based on their review.

### 2.5. Stage 2: Cultural Adaptation

The LLFDI was reconstructed to suit the Korean culture, and its validity and reliability were verified. In order to assess the cultural validity of the LLFDI, a group of experts was asked to evaluate the appropriateness of each item. In this study, we used the method of calculating the Item-level Content Validity Index (I-CVI) used in Polit and Beck’s study (2006) [22]. The experts were 15 professors/graduates/clinicians of occupational therapy. Details of inclusion criteria were given by an occupational therapist who has more than five years of clinical experience and a graduate student with a Ph.D. course who has experience in research related to the elderly. Additionally, they have lived in Korea for at least 25 years and received an education of Korean social culture according to Korea’s education law. Considering the appropriateness of each item when applied to the Korean culture, respondents were asked to respond in a 4-point Likert scale: “Very appropriate” (4 points), “Appropriate” (3 points), “Not appropriate” (2 points), and “Very inappropriate” (1 point). The I-CVI score calculation method divides the total points of an item into group numbers. For each item, if the I-CVI score was 0.78 or higher, the validity was considered appropriate, and items that obtained less than three points in the Likert scale were adjusted and recorded, as well as the reasons for the required corrections [23,24].

### 2.6. Stage 3: Reliability and Validity Analysis

A researcher visited 50 older adults living in the community and conducted an assessment to verify the reliability and validity of the reconstructed K-LLFDI after verifying its cultural feasibility with the expert group. However, in order to implement the reliability and validity verification, the number of subjects was insufficient to assume a normal distribution for the estimated coefficients. Therefore, using Bootstrapping in AMOS, we assume a normal distribution with an estimate of 100 iterations. As the K-LLFDI is a self-reported instrument, the researcher read the items to older adults who had vision problems. No data were dropped owing to errors after the assessment. Based on the collected data, internal consistency and construct validity were measured. This process is shown in Figure 1. Test–retest reliability is the ability of an instrument to obtain the same results over time when applied in a sample of stable individuals. The retest period was three weeks. Additionally, internal consistency was expressed as the Cronbach’s α value, and the higher the value, the greater the reliability [25]. In the field of social science, it is generally judged to be “acceptable” when it is 0.6 or higher, “good” when it is 0.7 or higher, and “very good when it is above 0.8 [19]. The commonly used Root Mean Square Error of Approximation (RMSEA), Good Fitness Index (GFI), Tucker–Lewis Index (TLI), and Comparative Fit Index (CFI) were used as methods to confirm model fit.

### 2.7. Statistical Analysis

Excel 2010 was used to compile the data collected in the survey and verify cultural validity. The results analysis and statistics were performed using SPSS statistical software and SPSS AMOS 25. All statistical significance levels were set at a *p*-value of 0.05.

## 3. Results

The study was conducted with older adults aged 65 years or more, with an average age of 79.24 years. Each average scores and standard deviations are as follows. Disability component (social role: 19.18 ± 6.681, personal role: 21.36 ± 4.685, instrumental role: 35.36 ± 11.933, management role: 11.58 ± 3.970), Function component (upper extremity: 31.56 ± 3.913, basic lower extremity: 57.04 ± 10.785, advanced lower extremity: 35.44 ± 13.135) (Table 1).

### 3.1. Cultural Adaptation

Based on the I-CVI evaluated by 15 experts, the value for item F23 in the Function component was 0.63. This item required adjustment, and based on the opinions of the group of experts (one occupational therapy professor and a researcher) F23 was adjusted as follows: “You can prepare for bed by opening a bed sheet or putting a blanket on the floor.” The I-CVI value obtained by the remaining items was 0.78 or higher, except for items F4, F7, and F30, which used the statement “Can run 0.8 km, 1.6 km, or more.” This statement was modified by the experts from 0.8 to 1 km and from 1.6 to 2 km before the K-LLFDI was finalized.

### 3.2. Reliability Analysis

#### 3.2.1. Test–Retest Reliability

The test–retest (Table 2) reliability assessment was conducted with 10 (20%) out of 50 older adults who participated in this study, compared to 14 (12%) out of 118 participants subject to re-test in the Brazilian version of the LLFDI performed by the study of De Almeida et al. (2016). The retest study comprised nine women and one man, and their average age was 80.8 years (SD ± 5.325). Test–retest reliability of the K-LLFDI was measured using the intraclass correlation coefficient (ICC). Regarding the Disability component, the ICC values were very high for the social role (0.948), the instrumental role (0.904), and the management role (0.964), while the ICC value for the personal role (0.795) was high. On the other hand, the ICC values of the Function component were very high for basic lower extremity (0.781) and advanced lower extremity (0.971) and moderate for upper extremity (0.565). Test–retest reliability of the K-LLFDI was measured and similar scores were obtained, with the exception of the upper extremity domain in the Function component, compared with the results of the original English version of the LLFDI (Jette, Haley, and Kooyoomjian, 2002) and the LLFDI-Br [20].

#### 3.2.2. Internal Consistency

Internal consistency is indicated by Cronbach’s α (Table 3). The overall internal consistency of the Disability component was high (α = 0.859), and the specific internal consistency per domain was as follows: social role (high; α = 0.862), personal role (satisfactory; α = 0.725), instrumental role (very high; α = 0.914), and management role (satisfactory; α = 0.771). On the other hand, the overall internal consistency of the Function component was very high (α = 0.965), and the internal consistency per domain was the following: upper extremity (satisfactory; α = 0.775), basic lower extremity (very high; α = 0.929), and advanced lower extremity (very high; α = 0.956). It is important to mention that the internal consistency results of the K-LLFDI were similar to Cronbach’s α values found for the original English version of the LLFDI (Jette et al., 2002) and the LLFDI-Br [20].

### 3.3. Validity Analysis

#### Construct Validity

To verify the construct validity of the K-LLFDI, a confirmatory factor analysis was conducted. Factor analysis results for the Disability component and its domains (social role, personal role, instrumental role, and management role), as well as for the Function component and its domains (upper extremity, basic lower extremity, and advanced lower extremity), are presented in Table 4 and Table 5. Regarding the Disability component, the following factor loadings were found to be below the generally accepted standard factor loading of 0.4: factor loading D14F of social role (0.326) factor loadings D4F and D8F of personal role (0.394 and 0.152, respectively); and factor loading D8L of management role (0.327). Similarly, in the Function component, the factor loading F3 (0.313) of upper extremity function and the factor loading F12 (0.393) of basic lower extremity function were slightly lower than the standard. All other items had factor loadings that were appropriate to the criteria. As a result, the average factor loading of the Disability and the Function components were 0.62 and 0.72, respectively, which is considered generally high. Overall, the model fit was not appropriate because the number of subjects was insufficient. (RMSEA = 0.114, TLI = 0.818, GFI = 0.898, CFI = 0.871).

## 4. Discussion

The purpose of this study was to perform cultural adaptation of the LLFDI and develop the Korean version of this instrument (K-LLFDI), assessing its reliability and validity. The purpose of this instrument is to properly assess the functions and disabilities of older adults living in communities. The study was conducted between December 2018 and February 2019 on 50 community-dwelling older adults aged 65 years or more who lived in the provinces of Gangwon and South Gyeongsang. The Korean version of the LLFDI was developed after the translation and cultural adaptation process of the English version of the LLFDI, and its reliability and validity assessment. The I-CVI calculation used by the group of experts during the cultural adaptation process of the K-LLFDI reported that only one out of 48 items of the K-LLFDI was not suitable, reflecting also the experts’ opinions. The item that needed to be adjusted was F23: “You can put a bed sheet into the mattress to prepare for bed”. The low score obtained for this item is attributed to the fact that most Korean older adults sleep on the ondol floor rather than on the bed because they adopt a traditional Korean house structure rather than a Western style. Additionally, the expert group recommended a change in the values used by the original version of the LLFDI in items F4, F7, and F30, from 0.8 to 1 km, and from 1.6 to 2 km, which were included in the revised items of the K-LLFDI. The original values might have been derived from the fact that the U.S. uses the mile unit whereas the km unit is used in Korea. Therefore, it was considered more appropriate to use 1 km or 2 km in the K-LLFDI so that older adults could better understand the intention of the item within the context of Korean culture. Except for item F23, all the remaining items had an I-CVI value of 0.78 or higher, and the overall adapted instrument had an I-CVI value of 0.95, which is considered to be highly relevant culturally. In a study by Haley et al. (2010), it was reported that language differences or cultural characteristics should be considered when using assessment tools developed in other cultures [26]. In this regard, the K-LLFDI developed and evaluated in this study is considered to have no limitations regarding cultural adaptation.

The test–retest reliability of the Disability component of the K-LLFDI was relatively high compared to the original English version of the instrument and the LLFDI-Br. As reported by the study of Maria et al. [19], it could be assumed that the personal characteristics of the subjects who participated during the assessment phase of the K-LLFDI might have affected the results owing to the relatively small number of items contained in the management role domain and the small number of people subject to retest (*n* = 10). However, the reliability of the upper extremity domain within the Function component was satisfactory (α = 0.565). In general, the retest reliability of the upper extremity domain in the K-LLFDI was very low, considering that reliability in the social sciences is considered acceptable with a level of 0.6 or higher. The original English version of the LLFDI and the Brazil version have a reliability of 0.912 and 0.87, respectively. There is the possibility that the body rhythms of participants might have affected the study’s results. In this regard, five subjects who participated in the retest assessment expressed that their performance varied considerably depending on their physical condition, weather, and quality of sleep. In order to obtain more objective results, researchers will need to ensure that the subjects are aware of these aspects.

The internal consistency of the items was expressed by Cronbach’s α. In the LLFDI-Br developed by Maria et al. (2016) results were similar for both the Disability (α = 0.889) and Function components (α = 0.948) [19]. Regarding the K-LLFDI, Cronbach’s α values in all the domains of the Disability and Function components was 0.7 or higher, indicating that the stability of the tool was high enough.

On the other hand, factor analysis was used to evaluate the configuration validity of the K-LLFDI. Results showed that the factor loading of D4F (“You can take care of the household”) was 0.394, while the factor loading of D8F (“You can take care of your own health”), a sub-area of the Disability component (personal role), was 0.152. Item D4F (“You can take care of the family life”) is considered to have had a great impact on Korean welfare services. Under South Korean laws, older adults aged 65 years or more can apply for housekeeping assistance services without much difficulty. As a result, it is assumed that some subjects did not perform their own household chores despite good functioning, which may have affected the study results. One participant answered in such a way that she received almost a perfect score in the Function component because of her physical condition. However, a person visited her at home three times a week to help her with cleaning and laundry. Regarding item D8F (“You can take care of your own health”), some older adults surveyed tended to give extreme answers during the examination because they thought their health conditions were excessively good, even free from disease, or were excessively bad. Extreme answers show that participants were not clearly aware of their health conditions, and this might have affected the factor loading. In the Function component, the factor loading of item F3 (“Can work off in long-sleeves”) in the upper extremity domain was 0.313, while the factor loading in the basic lower extremity domain was 0.393. As a result, both items did not fully reflect the upper area. Data coding showed that the average score of the two statements mentioned above was 4.6 and 4.76 out of 5 points, respectively, which is very high compared to the overall average score of 3.88 for the Function component. The reason for this score is not because the K-LLFDI directly observes the performance of the item but because it is an assessment tool that relies on the self-reports of subjects.

Some limitations of this study are mentioned as follows: the number of subjects surveyed for the reliability and validity assessment was small, there were a greater proportion of women than men, and the self-reporting assessment can make a substantial difference from the actual level of function. Therefore, it is believed that the model fit was very low. Further research requires larger sample sizes and, in addition, a visual aid should be provided during the evaluation process to assist the subjects in understanding the questions contained in the original English version manual of the LLFDI to encourage them to answer more objectively.

## 5. Conclusions

This study aimed to perform the cultural adaptation of the LLFDI in order to develop the Korean version of this instrument (K-LLFDI) and verify its reliability and validity. Overall, each component was well reflected by the sub-items. The K-LLFDI is expected to be instrumental in solving the rapidly growing problems of community-dwelling older adults, considering that this instrument has been rarely used in Korea. It can be used in the clinical environment for occupational therapy to evaluate functions and disabilities regarding the performance of everyday life in older adults. Moreover, it is expected to be instrumental in solving the rapidly growing problems of community-dwelling older adults. However, further research is required, and practical applications should be developed.

## Figures and Tables

**Table 1:** K-LLFDI mean score and standard deviation per component (*n* = 50).

Categories		M(SD)
Disability component	Social role	19.18(6.681)
	Personal role	21.36(4.685)
	Instrumental role	35.36(11.933)
	Management role	11.58(3.970)
Function component	Upper extremity	31.56(3.913)
	Basic lower extremity	57.04(10.785)
	Advanced lower extremity	35.44(13.135)

Note. K-LLFDI = Korean version of the Late-Life Function and Disability Instrument; M = mean; SD = standard deviation.

**Table 2:** Test–retest reliability of the K-LLFDI (*n* = 50).

Test–Retest, ICC [95% CI]				
Domain	Original	Swedish	Brazilian	Korean
	Disability component			
Frequency				
Social role	0.753	0.82	0.81 [0.52, 0.93]	0.948 [0.816, 0.987]
Personal role	0.633		0.53 [0.00, 0.82]	0.795 [0.397, 0.944]
Limitation				
Instrumental role	0.833	0.91	0.59 [0.12, 0.85]	0.904 [0.680, 0.975]
Management role	0.435		0.27 [0.00, 0.70]	0.964 [0.871, 0.991]
	Function component			
Upper extremity	0.912	0.87	0.95 [0.86, 0.98]	0.565 [-0.025, 0.869]
Basic lower extremity	0.976	0.89	0.93 [0.81, 0.98]	0.781 [0.366, 0.940]
Advanced lower extremity	0.966	0.91	0.78 [0.44, 0.93]	0.971 [0.895, 0.993]

Note. CI = confidence interval; ICC = Intraclass correlation coefficient; LLFDI = Late-Life Function and Disability Instrument.

**Table 3:** Internal consistency of the K-LLFDI.

Internal Consistency, Cronbach’s α				
Domain	Original	Swedish	Brazilian	Korean
	Disability component			
Frequency				
Social role	0.80	0.90	0.807	0.862
Personal role	0.73		0.601	0.725
Limitation				
Instrumental role	0.92	0.95	0.856	0.914
Management role	0.63		0.611	0.771
	Function component			
Upper extremity	0.912	0.87	0.819	0.775
Basic lower extremity	0.976	0.89	0.889	0.929
Advanced lower extremity	0.966	0.91	0.914	0.956

**Table 4:** Factor analysis for the Disability component of the K-LLFDI four domains.

Disability Component		
Domain	Item No.	Factor Loading
Social role	D1F	0.440
	D2F	0.533
	D3F	0.489
	D5F	0.901
	D6F	0.785
	D9F	0.780
	D11F	0.898
	D12F	0.620
	D14F	0.326
Personal role	D4F	0.384
	D7F	0.891
	D8F	0.152
	D10F	0.429
	D13F	0.506
	D15F	0.690
	D16F	0.460
Instrumental role	D2L	0.797
	D3L	0.624
	D4L	0.519
	D5L	0.905
	D6L	0.875
	D9L	0.771
	D10L	0.714
	D12L	0.651
	D13L	0.422
	D14L	0.469
	D15L	0.770
	D16L	0.400
Management role	D1L	0.466
	D7L	0.936
	D8L	0.327
	D11L	0.904

Note. Values > 0.400 indicate that the item has adequate factor loading.

**Table 5:** Factor analysis for the Function component of the K-LLFDI with three domains.

Function Component		
Domain	Item No.	Factor Loading
Upper extremity	F1	0.543
	F3	0.313
	F5	0.769
	F6	0.575
	F13	0.469
	F16	0.619
	F17	0.798
Basic lower extremity	F2	0.716
	F10	0.445
	F11	0.762
	F12	0.383
	F14	0.812
	F15	0.626
	F18	0.801
	F21	0.703
	F22	0.866
	F23	0.610
	F25	0.698
	F26	0.808
	F28	0.790
	F31	0.835
Advanced lower extremity	F4	0.854
	F7	0.785
	F8	0.814
	F9	0.931
	F19	0.858
	F20	0.964
	F24	0.852
	F27	0.637
	F29	0.772
	F30	0.827
	F32	0.730

Note. Values >0.400 indicate that the item has adequate factor loading.

## Data Availability

The data that support the findings of this study are available on request from the corresponding author, [H.Y.P.]. The data are not publicly available due to restrictions (e.g., they contain information that could compromise the privacy of research participants).
